# Pulmonary Tuberculosis

**Published:** 1889-07

**Authors:** 


**Pulmonary Tuberculosis.** Its Etiology, Symptomatology, and Therapeutics. By PROF. DR. H. VON ZIEMSSEN, Director of the Medical Clinic at Munich. Translated by David J. Doherty, A. M., M. D., Instructor in the Chicago Polyclinic. 12mo; pp. 119. Price, twenty-five cents. Detroit: George S. Davis. 1888.

This volume is one of the Physician's Leisure Library, and contains the latest views of the teachers of Europe on Pulmonary Tuberculosis. In corresponding parts, it discusses the etiology, diagnosis, and treatment. There is also an appendix containing—(*a*) Tuberculosis in American Prisons, and (*b*) Method of Examining Sputum for Bacilli. It will be thus seen that we have here, in a very convenient form, an important contribution on a vital question, and in this connection we would refer our readers also to a paper by Dr. Hermann M. Biggs on this subject, recently published in this journal. The activity in the study of tuberculosis has steadily increased since Koch's famous discovery, and we look forward to the time when the results of treatment will be much better than they are now. Small volumes, like this, increase the general information by going where larger ones could not, and are therefore of much advantage. The translation is good, though, as Dr. Doherty points out, there is some diffuseness and many repetitions which could not be avoided, as the matter was delivered in the form of lectures. This little book deserves a wide reading.

